# Morphological Assessment of Stage HH38 of the Japanese Quail (*Coturnix japonica*) Heart by Micro-Sonogram

**DOI:** 10.3390/mps9030071

**Published:** 2026-05-02

**Authors:** Jaden Roe, Ashlyn Benavides, Michael B. Filla, Douglas C. Bittel, Whitney Shae, Geetha Haligheri, James E. O’Brien, Nataliya Kibiryeva

**Affiliations:** 1 Department of Biomedical Sciences, Kansas City University, Kansas City, MO 64106, USA; jaden.roe@kansascity.edu (J.R.); ashlyn.benavides@kansascity.edu (A.B.); mfilla@kansascity.edu (M.B.F.); wshae@kansascity.edu (W.S.); 2 Ward Family Heart Center, Children’s Mercy Hospital, Kansas City, MO 64108, USA; ghaligheri@cmh.edu (G.H.); jobrien@cmh.edu (J.E.O.J.)

**Keywords:** echocardiography, cardiovascular embryogenesis, quail (*Coturnix japonica*), doppler

## Abstract

A challenge of studying mammalian cardiac embryogenesis is the limited ability to perform experimental manipulations in animal models. The avian embryo is widely accepted as a model for mammalian heart developmental studies. In this study, we establish the methodology and protocols for studying the Japanese quail (*Coturnix japonica*) heart at embryonic day 10 (HH38) using the FUJIFILM VisualSonics Vevo 3100 ultrasound system equipped with a MX550D small animal cardiology transducer. These protocols were designed to measure right ventricular wall thickness, pulmonary artery diameter, and the outflow velocities of the right ventricular outflow tract (RVOT) and the pulmonary artery (PA), thereby establishing baseline parameters of the normally developing quail morphology. Quail embryos are an ideal model for cardiovascular research due to their short incubation period (16–17 days), experimental accessibility, and strong similarities to mammalian heart development. These developmental similarities include, but are not limited to, looping, chamber septation, and the development of a true four-chamber heart. High-resolution imaging modalities, including ultrasound and optical coherence tomography, enable noninvasive, real-time visualization of cardiac morphology and function throughout development. Echocardiography allows for quantitative and qualitative assessments of myocardial structure and cardiac hemodynamics. The similarity to the mammalian heart, combined with rapid embryogenesis, makes quail embryos a valuable model for investigating congenital heart defects, genetic modifications, and fundamental cardiac developmental processes. In this study, we describe reproducible incubation protocols and baseline echocardiographic parameters used to evaluate morphological and physiological changes in the developing embryonic quail heart on embryonic day 10.

## 1. Introduction

Cardiogenesis is a highly coordinated developmental process in which a simple linear heart tube undergoes a series of structural and functional changes to form a fully developed four-chambered heart. This transformation involves cardiac looping, chamber formation, septation, and outflow tract development, all of which are regulated by tightly controlled genetic programs and biomechanical forces [1,2,3]. Because these processes occur in a precise and sequential manner, even small perturbations can alter cardiac structure or function. For this reason, establishing reliable methods to visualize and quantify normal cardiac development is essential for understanding how the heart forms.

Animal models have been critical for studying cardiogenesis. While mammalian systems offer strong clinical relevance, they are often limited by accessibility, longer gestational timelines, and increased cost and regulatory constraints. In contrast, avian embryos provide a practical and accessible alternative that allows for direct visualization and manipulation throughout development.

The Japanese quail (*Coturnix japonica*) offers several advantages as a model for developmental studies, including a short incubation period, ease of handling, and compatibility with experimental manipulation. Most notably, quail heart organogenesis follows a similar embryonic pattern to that of the mammalian heart, including the formation of a true four-chambered heart [4,5]. Embryonic development is rapid, with a total incubation period of 16–17 days, and a distinct four-chamber anatomy evident by embryonic day 10. Importantly, the fundamental processes of heart development in avian species closely parallel those observed in mammals, including cardiac looping, chamber septation, etc. The accessibility, low cost, and short developmental timeline of the quail model enable efficient, large-scale analysis of cardiac morphogenesis [6,7]. Additionally, the quail heart is readily accessible for echocardiographic visualization.

Japanese quail are a well-established avian model for chorioallantoic membrane (CAM)-based research [7]. The CAM permits direct visualization of the developing embryo, while maintaining intact vascular structures, thereby preventing exsanguination during imaging. This feature of the quail model enables prolonged visualization and repeated echocardiographic assessment of the embryonic heart.

Echocardiography provides a noninvasive and translationally relevant method for evaluating cardiac structure and hemodynamics in real time [8]. This technique allows for the measurement of myocardial dimensions, vascular structures, and blood flow velocities, linking morphology with function during development. Given its widespread use in clinical cardiology, echocardiography also offers a practical bridge between experimental models and human cardiac physiology.

Previous work has documented the use of echocardiographic analysis in the chicken heart. However, several of these papers seek to describe the developmental process of the heart over a longitudinal period. One such example is McQuinn and colleagues [9] where they described the organogenesis of the developing heart throughout stages 9–39 of chick embryo development.

We propose the Japanese quail (*Coturnix japonica*) as an effective alternative cardiovascular developmental model at the stage where we would expect to see morphological differences if various cardiogenic pathways were to be genetically altered. We sought to optimize methods and protocols for the repetitive micro-sonography of quail embryos in vivo. Despite these advantages, there remains a lack of standardized protocols for quantitatively assessing cardiac morphology and function in the quail embryo.

Using the Fujifilm VisualSonics Vevo 3100 ultrasound system, we performed real-time echocardiographic imaging of the quail heart on embryonic day 10 (HH38). Embryonic day 10 was chosen because it is the last and the largest stage that can be imaged using micro-sonography. Additionally, at this stage the heart has progressed through the major stages of morphogenesis and presents with clearly defined features that can be quantitatively assessed. Two-dimensional measurements and Doppler assessments were used to quantify cardiac outflow velocities, ventricular wall thickness, and pulmonary arterial diameter. Many scans were performed, the majority of which were used to train our team and to determine which protocols should be followed to produce optimal scans. The scans with the best visualization of anatomical features were then analyzed to establish the baseline morphological and functional parameters of the normally developing quail heart. By defining baseline morphological and functional parameters at this stage, this work provides a framework for future studies aimed at examining normal cardiogenesis as well as experimentally induced developmental changes, including genetic knockdown studies investigating the developmental origins of congenital heart defects.

## 2. Methods and Procedures

All procedures were conducted in accordance with institutional guidelines and AVMA Guidelines for the Euthanasia of Animals. In accordance with Section 3.4.4 of the AVMA Guidelines, all quail embryos were decapitated at stage HH38 on day 10 of incubation.

### 2.1. Procurement and Incubation of Quail Eggs

Fertilized quail eggs were purchased from Ozark Egg Company, Stover, MO. Early incubation efforts targeted approximately 40 eggs per cohort to support imaging throughput with an expected incubation success rate of 70%. Subsequently, a twice-weekly incubation schedule was adopted, with 20 eggs set for incubation on Mondays and Tuesdays to allow for completion of incubation on the following Thursday and Friday, corresponding to 10 days of embryonic development. This was performed over a month-long period, correlating to ~160 eggs incubated. This large imaging throughput was initially necessary to adapt to the minuscule manipulations required for the proper visualization of the quail embryos. Due to this, many of the early images did not correspond to visualized structures usable for data acquisition. However, following this initial period, the echocardiographic experience of the primary investigator, guided by the pediatric cardiologist, was sufficient to obtain imaging that was consistent with scans capable of data analysis.

An incubation period of 10 days was selected to allow the longest developmental period before visualization. Additionally, the longer development was chosen as, at this stage, we would expect the manifestation of congenital heart defect abnormalities. This stage was also chosen due to developmental constraints associated with quail embryogenesis. Japanese quail begin feather development at approximately embryonic day 9, with substantial feather growth continuing after day 10, impairing visualization [6]. During experimentation, we observed that feather formation significantly impairs echocardiographic visualization of the developing heart by introducing artifacts and acoustic shadowing, thereby reducing image quality and data reliability.

To control for confounding variables, the fertilized eggs were homogeneously distributed on an incubation tray in a Genesis model 1588 (Hova-Bator Incubator, Savannah, GA, USA), maintained at 38 °C. Each egg was marked on the superior surface to ensure consistent orientation during post-incubation access. Throughout the incubation period, distilled deionized water was added to the chamber’s reservoir to maintain appropriate humidity levels for proper growth conditions.

### 2.2. Quail Embryo Preparation for Echocardiography

**Figure 1:**
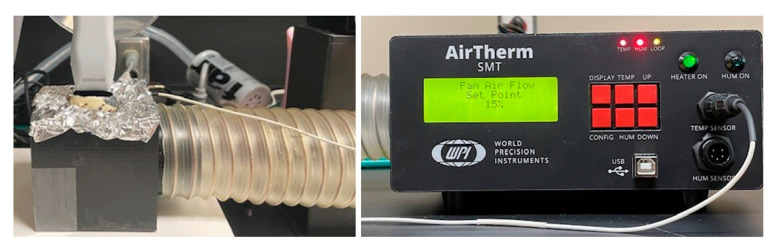
3D-printed imaging incubator with warm air circulated underneath.

Preparation of the quail embryos for echocardiography required careful temperature control throughout the procedure to preserve embryonic viability. Although embryos were humanely terminated in accordance with institutional and AVMA regulatory guidelines following imaging, it was essential to maintain normothermic incubation-like temperatures during preparation and scanning. To achieve this, several procedures were performed as follows. Eggs not actively being prepared or scanned were kept in the incubator until use. Upon removal for shell preparation, eggs were immediately placed on a heating pad. During echocardiographic imaging, temperature was maintained by circulating warm air through a custom 3D-printed imaging incubator, which supported the egg in an aluminum foil cradle with heated airflow beneath it by the Airtherm SMT Model 98736-3 heater (World Precision Instruments, Sarasota, FL, USA) (Figure 1). A temperature sensor was secured underneath the aluminum foil cradle to monitor heating throughout the course of imaging.

**Figure 2:**
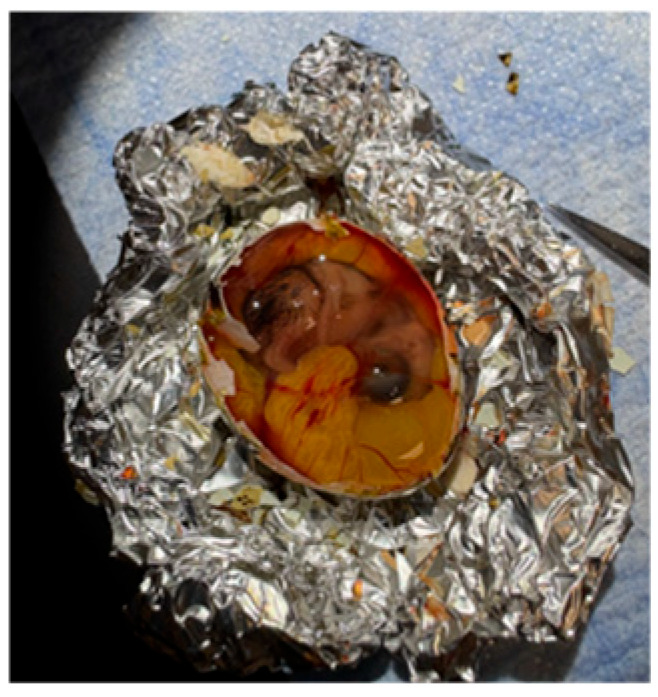
Quail shell removal set-up with quail egg placed in an aluminum foil cradle, with a heating pad underneath, and a light aimed directly on the superior aspect of the egg.

Egg shell removal was performed on a heating pad under direct illumination to enhance visualization (Figure 2). A molded aluminum foil cradle was used to stabilize the egg and prevent its rolling during manipulation. The peeling process was initiated by gently tapping the inferomedial, wider aspect of the egg with the blunt end of a dull instrument, just below the reference line on the superior aspect of the egg marked during incubation. This initial opening facilitated a controlled shell removal process using forceps. Care was taken to avoid damaging the thick outer membrane beneath the shell, which is adherent to the underlying chorioallantoic membrane containing the embryo’s vasculature, to prevent rupture of these vessels and resultant exsanguination. A visualization window approximately 3 cm × 2 cm, making up most of the superior aspect of the egg, was created. The outer membrane was then carefully separated from the chorioallantoic membrane, as disruption of the latter results in rapid exsanguination and significantly diminishes the duration of viable imaging. Small tears in the outer membrane were used as initiation points to facilitate this controlled separation process.

Following shell and membrane removal, the egg was prepared and positioned for echocardiographic imaging. A double-layered aluminum foil insert was secured within the 3D-printed incubator, and the prepared egg was positioned upon this heated opening. A square sheet of plastic wrap was placed tautly over the visualization window to form a stable imaging interface for the echo probe. Gauze was placed between the foil borders and the positioned egg to further stabilize the egg and prevent its shifting during scanning. Throughout imaging, 38 °C air was continuously circulated beneath the foil cradle to maintain appropriate temperature conditions.

### 2.3. Echocardiographic Imaging of Quail Embryos

Echocardiography can provide comprehensive information on cardiac anatomy, physiology, and mechanical properties. In humans, echocardiography consists of transmitting high-frequency sound waves, which are reflected off different tissue moieties (e.g., myocardium, blood, valves, etc.) back to an ultrasound transducer that receives the reflected signal off all tissues. Software dynamically processes the incoming signals from the different tissues and generates a real-time image based on the known acoustic impedances of each tissue. B-mode produces 2-D views of the heart (short- or long-axis), allowing the assessment of cardiac chamber dimensions, physiology, and visualization of cardiac anatomic structure such as papillary muscle and valves.

Using the Fujifilm Visual Sonics Vevo 3100 ultrasound system (FUJIFILM VisualSonics Inc., Bothell, WA, USA), real-time echocardiographic imaging of the quail heart on embryonic day 10 (HH38) was performed. The MX550D transducer was utilized for image acquisition, which is the gold standard for small animal model micro-sonography. The transducer has an axial resolution of 40 µm, lateral resolution of 80 µm, center frequency of 40 MHz (broadband 25–55 MHz) and a depth of up to 15 mm. We used 3 main modes of echocardiography: brightness mode (B-mode) which produces 2-dimensional (2D) views of the heart (long-axis or short-axis) and Doppler echocardiography which included both color Doppler and pulse wave Doppler (PWD). For 2D images the frequency was 40–60 MHz, with frame rates ranging from 150 to 200 frames per second (fps) with a gain of 25–30 dB and a dynamic range of ~50 dB. For color Doppler the frequency was 30 MHz, 20–30 fps frame rate, and a pulse repetition frequency (PRF) of 10–15 kHz. For the PWD, the frequency was 30 MHz, dynamic range was 30 dB and PRF was 25 kHz. During PWD assessment we only selected the images where the Doppler was placed in line with the blood flow, aiming for an angle of insonation that is less than 40° and therefore angle correction was not required for any of the images chosen.

To begin, a small amount of ultrasound gel, the size of a quarter, was applied to the plastic wrap covering the visualization window and evenly distributed using the echo probe to ensure proper acoustic coupling. The imaging depth was ~14 mm. Quail embryo heart rates are extremely fast, around 600–750 bpm, when compared to the heart rate of human embryos, i.e., 150–170 bpm. Multiple scans were performed on each embryo to obtain consistent morphological and functional data. Gain and depth settings were conservatively modified throughout the course of imaging and across embryos. Like humans, there were variances across the embryos that required subtle manipulation of gain/depth to maximize morphological structure visualization.

Scans were acquired in both long-axis and short-axis views. The long-axis view provided a detailed visualization of cardiac morphology, allowing measurement of myocardial thickness, right ventricular outflow tract and pulmonary outflow velocities, and pulmonary arterial diameter. The short-axis view allowed for additional measurement of the myocardial thickness, as well as primary measurement of right ventricular outflow tract velocity. Attempts to utilize the M-Mode function of the Vevo 3100 system were unsuccessful. M-Mode is universally utilized to quantify cardiac function. However, the size of the quail embryonic heart and the rapid heart rate limited the reproducibility of this parameter. Future work will seek to incorporate this imaging modality and standardize its implementation.

The process of scanning was initiated by lowering the echo probe gently onto the embryo’s surface to calibrate the image on the Vevo 3100 system. All imaging was performed with the echo probe perpendicular to the surface of the embryo. Further manipulation was needed to obtain the long-axis view, which served as an anchor image for the rest of the scanning process. The scan selection was based on visualization of specific landmarks (pulmonary artery (PA) being the anterior outflow and aorta being the posterior outflow) rather than relying on signal-to-noise ratio. At the long-axis view, a B-Mode scan was performed to visualize all the relevant anatomical structures, such as the cardiac chambers and outflow tracts. The probe was then adjusted to prioritize visualization of the ventricular wall to measure the ventricular outflow dimensions, followed by Doppler. However, the visualization of the ventricular outflow in the long-axis was sometimes inconsistent. The probe was further manipulated cranially along this same axis to visualize the pulmonary artery, allowing B-Mode measurement of diameter and Doppler assessment of outflow velocity.

Rotation of the echo probe 90° from the long-axis view provided the short-axis view. From this view, fine adjustments were required to frame the heart properly. Following visualization of this view, both B-Mode and Doppler scans were obtained to provide analysis for ventricular wall thickness and ventricular outflow tract velocity, respectively. The Doppler probe on the Vevo 3100 screen was localized to the right ventricle for outflow tract velocity measurement during scanning. All processes and steps were attempted on each viable embryo. However, not every embryo showed sufficient visualization of each desired feature. This is due to expected variations in where the embryo was sitting, or if the shell was blocking visualization.

Due to the small size of the quail embryos, with structures often less than 0.5 mm thick, precise echo probe manipulation was critical. To improve operator skill and efficiency, 2–4 chicken embryos were incubated and scanned as practice models alongside the quail embryos but were not utilized for data collection. Familiarity with human echocardiographic procedures, as well as consultation with a pediatric cardiologist, facilitated efficient imaging on the Vevo 3100 system due to the inherent ability of not having to reposition the probe for every scan.

### 2.4. Quantification of Embryonic Cardiac Morphology and Function

In totality, around 100 embryo scans were analyzed for morphological assessment. For each morphological feature, the 10 highest quality B-Mode scans were selected for analysis. An objective system was used to determine “highest quality” by most prominent visualization of cardiac morphological structures of interest. This objective system was informed by collaboration with a pediatric cardiologist with the primary criteria being visualization of cardiac structures based on specific anatomical landmarks such as the developing lungs, ventricular outflow tracts, great vessels, etc. Each embryo was scanned to visualize as many morphological features as possible. However, due to variability across embryos and time restrictions, most embryos allowed for analysis of one morphological feature.

Due to this, 10 measurements of each morphological structure were nested in each embryo and performed across 10 embryos per feature, totaling 100 data points for all ventricular wall thicknesses and pulmonary artery diameter measurements. These measurements were taken from one hyperechoic edge to the opposing hyperechoic edge, in both systole and diastole. Because right ventricular wall thickness was visualized in both long- and short-axes, this process was performed in the long-axis and repeated upon manipulation to the short-axis. Pulmonary artery thickness was measured using the same procedure in the long-axis only. For each outflow velocity Doppler analysis, 3–7 measurements were nested in each embryo and performed across 10 embryos. Pulmonary artery outflow velocity was measured distal to the right ventricle in the long-axis view, whereas right ventricular outflow tract velocity was measured in the center of the right ventricular chamber in the short-axis view. All measurements were performed by a single observer and intra-observer error is reported in the results section.

For each site, the mean (SD), median, and IQR range (minimum–maximum) were calculated to summarize the distribution of measurements. In addition, we fit a linear mixed-effects model for each assessment with random intercepts for subject to assess site-specific measurement repeatability using intraclass correlation coefficients (ICC) and measurement variability with the coefficient of variation (CV).

## 3. Results

### 3.1. Echocardiographic Visualization of the Embryonic Quail Heart

**Figure 3:**
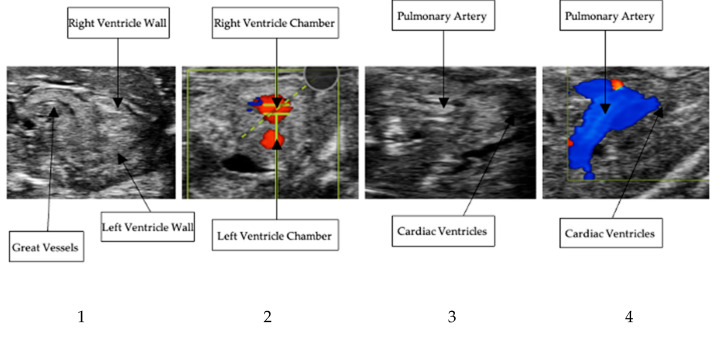
From **left** to **right**: (**1**. long-axis B-Mode localized at ventricles, **2**. short-axis Doppler of the right ventricle outflow tract, **3**. long-axis B-Mode localized at the pulmonary artery, **4**. long-axis Doppler of the pulmonary artery outflow tract).

High-resolution echocardiographic imaging enabled consistent visualization of the embryonic quail heart at embryonic day 10. Both long-axis and short-axis views were successfully acquired, allowing visualization of the right ventricular wall, pulmonary artery, and associated outflows. For all Doppler imaging, blue indicates flow moving away from the probe. Whereas, red indicates flow moving towards the probe. Representative B-Mode and Doppler images of various scans are presented in Figure 3.

### 3.2. Long-Axis Right Ventricular Wall Thickness

**Figure 4:**
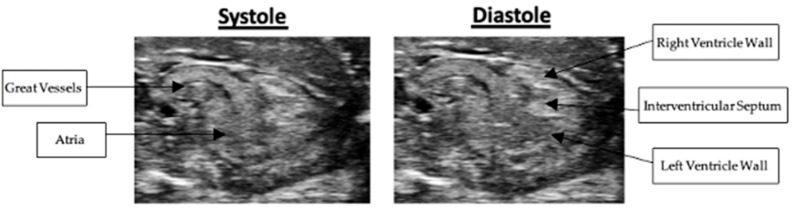
Systolic and diastolic long-axis B-Mode scans of right ventricular wall thickness.

**Figure 5:**
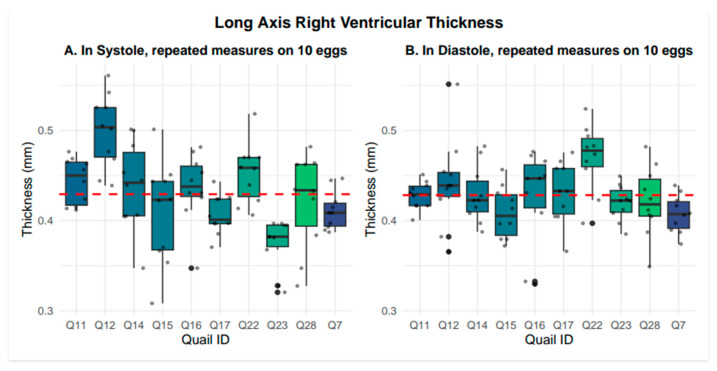
Nested box-and-whisker plots showing long-axis right ventricular thickness, in both systole and diastole, across 10 embryos with 10 nested measurements per embryo, where the grand mean is shown with the red dashed line. Systole averaged 0.43 mm +/− 0.05 mm, while diastole averaged 0.43 mm +/− 0.04 mm.

Right ventricular wall thickness was measured in both long- and short-axis views. In the long-axis view, right ventricular wall thickness averaged 0.43 +/− 0.05 mm in systole and 0.43 +/− 0.04 mm in diastole. Representative systolic and diastolic long-axis images are presented in Figure 4. Nested box-and-whisker plots for long-axis right ventricular wall thickness in both systole and diastole are provided in Figure 5. Descriptive and reliability statistics for long-axis right ventricular wall thickness are provided in Table 1. The ICC represents the proportion of variance attributable to between-subject differences. Residual standard deviation is the absolute measurement variability in the original units, while the CV quantifies the relative variability by expressing the residual standard deviation as a percentage of the mean.

### 3.3. Short-Axis Right Ventricular Wall Thickness

**Figure 6:**
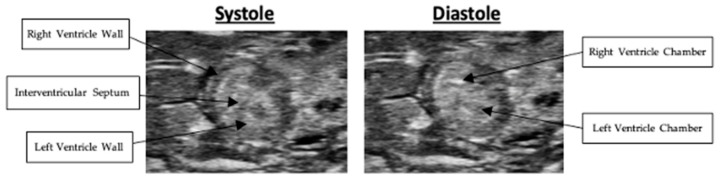
Systolic and diastolic short-axis B-Mode scans of right ventricular wall thickness.

**Figure 7:**
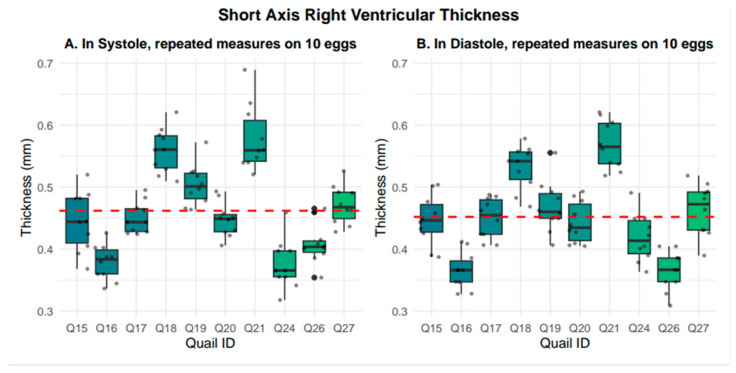
Nested box-and-whisker plots showing short-axis right ventricular thickness, in both systole and diastole, across 10 embryos with 10 nested measurements per embryo, where the grand mean is shown with the red dashed line. Systole averaged 0.46 mm +/− 0.07 mm, while diastole averaged 0.45 mm +/− 0.07 mm.

In the short-axis view, right ventricular wall thickness averaged 0.46 +/− 0.07 mm in systole and 0.45 +/− 0.07 mm in diastole. Representative systolic and diastolic short-axis images are presented in Figure 6. Nested box-and-whisker plots for short-axis right ventricular wall thickness in both systole and diastole are provided in Figure 7. Descriptive and reliability statistics for short-axis right ventricular wall thickness are provided in Table 2.

### 3.4. Pulmonary Artery Diameter

**Figure 8:**
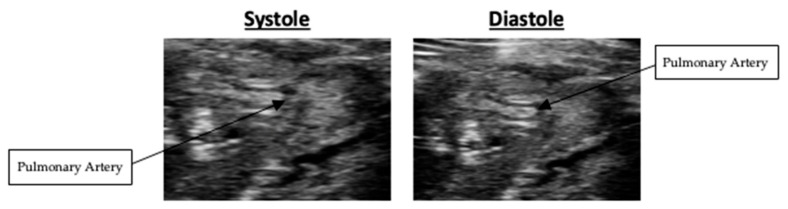
Systolic and diastolic long-axis B-Mode scans of pulmonary artery diameter.

**Figure 9:**
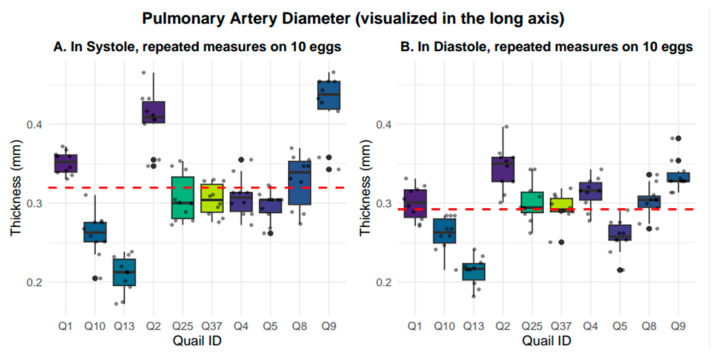
Nested box-and-whisker plots showing long-axis pulmonary artery diameter, in both systole and diastole, across 10 embryos with 10 nested measurements per embryo, where the grand mean is shown with the red dashed line. Systole averaged 0.32 mm +/− 0.07 mm, while diastole averaged 0.29 mm +/− 0.04 mm.

Pulmonary artery diameter was measured in the long-axis view. The pulmonary artery diameter averaged 0.32 +/− 0.07 mm in systole and 0.29 +/− 0.04 mm in diastole. Representative systolic and diastolic images are presented in Figure 8. Nested box-and whisker-plots for long-axis pulmonary artery diameter in both systole and diastole are provided in Figure 9. Descriptive and reliability statistics for short-axis right ventricular wall thickness are provided in Table 3.

### 3.5. Right Ventricular Outflow and Pulmonary Artery Velocity

**Figure 10:**
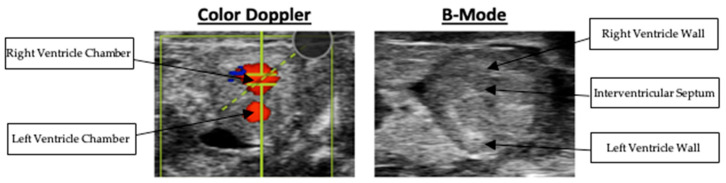
Color Doppler and B-Mode scans of right ventricular outflow tract velocity.

**Figure 11:**
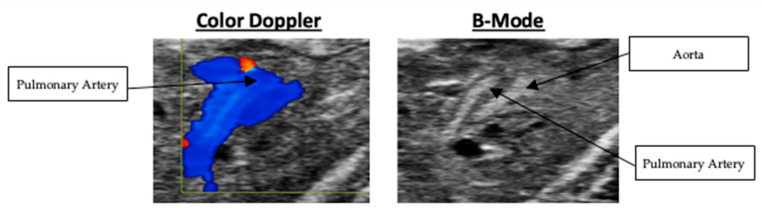
Color Doppler and B-Mode scans of pulmonary artery outflow velocity.

**Figure 12:**
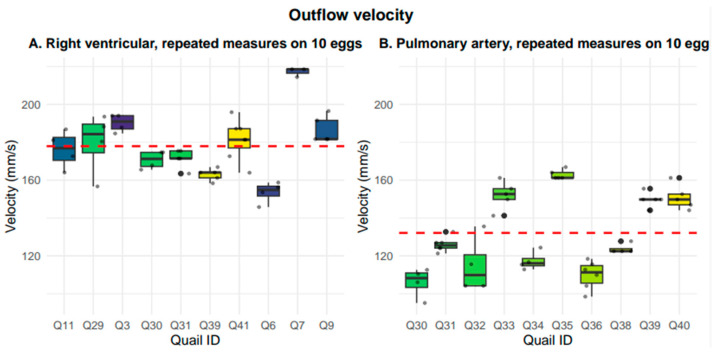
Nested box-and-whisker plots showing short-axis right ventricular outflow tract (RVOT) velocity and long-axis pulmonary artery outflow tract velocity across 10 embryos with 3–7 nested measurements per embryo, where the grand mean is shown with the red dashed line. The average outflow velocity for RVOT velocity was 177.9 mm/s +/− 16.48 mm/s and the average outflow velocity for pulmonary artery outflow was 132.1 mm/s +/− 20.7 mm/s.

Right ventricular outflow velocity was measured in the short-axis view. The right ventricular outflow velocity averaged 177.92 +/− 16.48 mm/s. Although Doppler was used to obtain this measurement, the respective Doppler and B-Mode imaging are presented in Figure 10. Pulmonary artery velocity was measured in the long-axis view. The pulmonary artery velocity averaged 132.14 ± 20.66 mm/s. The respective Doppler and B-Mode imaging are presented in Figure 11. The nested box-and-whisker plot for both right ventricular outflow tract and pulmonary artery outflow velocity is provided in Figure 12. Descriptive and reliability statistics for right ventricular outflow velocity and pulmonary artery outflow velocity are provided in Table 4.

Repeatability differed by morphology and function. Pulmonary artery velocity (ICC = 0.899) and right ventricular outflow velocity (ICC = 0.805) demonstrated high reliability, while pulmonary artery diameter in systole (0.832) and diastole (0.750), along with short-axis right ventricular wall thickness in diastole (0.746) and systole (0.776), showed moderate repeatability. In contrast, long-axis right ventricular wall thickness in diastole (ICC = 0.390) and systole (ICC = 0.137) exhibited lower reliability. Coefficients of variation ranged from 4.6% to 9.1%, indicating low measurement variability for all morphology and functions. Collectively, these measurements establish baseline morphological and functional parameters of the normally developing embryonic quail heart at embryonic day 10.

## 4. Discussion

This study establishes the Japanese quail (*Coturnix japonica*) as a viable animal model for the echocardiographic assessment of embryonic cardiac morphology and function. Utilizing high-resolution ultrasound imaging, we successfully obtained reproducible long- and short-axis views of the quail embryo heart at embryonic day 10. We also quantified significant structural and hemodynamic parameters, including right ventricular wall thickness, pulmonary artery diameter, and cardiac outflow velocities. Collectively, these measurements provide a substantial baseline framework for the normally developing quail heart.

Notably, avian species, including quail, have been reported to exhibit a higher incidence of sporadic congenital heart defects compared to humans. This characteristic further strengthens the utility of the quail model for investigating the etiology of these defects. In this study, consultation with a pediatric cardiologist allowed for the identification of echocardiographic features that were suggestive of structural defects within several scans. Specifically, they determined various embryos to contain abnormalities ranging from ventricular septal defects (VSD) to abnormal outflow patterns. Although systematic classifications of congenital heart defects are beyond the scope of this work, these observations highlight the feasibility of detecting and characterizing congenital defects in the developing quail embryo at the HH38 stage utilizing the protocols outlined here.

In a broad sense, the objective of this research is to establish a comparable morphological parameter set to support future studies investigating the molecular regulation of cardiogenesis. Our laboratory focuses on the function of the spliceosomal apparatus, and the role it plays in organogenesis of the heart, with particular interest in a subset of small non-coding RNAs known as Small Cajal body-associated RNAs (scaRNAs) and their role in the regulation of this apparatus. Our future studies will utilize targeted genetic knockdown approaches to disrupt specific scaRNAs in the developing quail embryos to ascertain their effect on cardiogenesis. Using the echocardiographic protocol and baseline morphological parameters established here, future studies will seek to compare echocardiographic data of genetically modified quail embryos to the morphological framework established in this study. Deviations in ventricular wall thickness, vascular dimensions, or flow velocities would provide the baseline for the support of an association between these genetic manipulations and congenital heart defects.

Although this study establishes the first baseline morphological parameter set, it is not without limitations. Measurements were obtained at a single developmental period, constraining the application to a single point in time. However, the HH38 stage is the last stage that can be visualized via micro-sonography; thus, this is the stage that we would most likely see the manifestation of developmental abnormalities in future studies. Further optimization implementing shell-less (Egg-in-cube) “in cubo” methods will allow for longitudinal optical coherence tomography and micro-sonogram studies of the same embryo over multiple stages [10]. This will allow for the description of the quail heart at different stages of development, which will be useful to describe the various processes that contribute to the manifestation of mature morphological features. While outside of the scope of this study, longitudinal assessment across multiple embryonic stages will be possible using this “in cubo” system and will lead to further understanding of these morphological processes by allowing for manipulation and imaging at various stages. We would expect to see the developmental patterns mentioned previously, as well as a longitudinal development of the structures analyzed in this paper.

Additionally, the utilization of a modeling algorithm for the measurement of these morphological parameters would help strengthen the uniform methodology of data collection. This was not performed due to time restrictions and restrictions of the Vevo 3100 system itself. The function that would have had the highest data yield was M-Mode mapping. This allows for the mapping of the ventricular chamber movement, in the short-axis, to gather data on ejection fraction, wall thickness, etc. During our study, we found it incredibly challenging to gather this data due to the increased hyperechoic presentation in the chamber itself that disrupted the measurement. This could have been due to human error or instrumentation, but the proper use of the M-Mode feature would be worth investigating to gather these measurements. Despite these limitations, the present work still provides a critical methodological and quantitative foundational parameter set. Future genetic and developmental studies will be able to use the methods and protocols established here to analyze morphological features of the developing quail heart.

In summary, this study demonstrates that high-resolution echocardiography is a viable method for reliable, quantitative assessment of the embryonic cardiac morphology of the Japanese quail. The baseline parameter set established here supports the use of this model and establishes the methods and protocols for future investigations into the molecular and genetic origins of various congenital heart defects and reveals the developing quail embryo as a powerful model for the investigation of translational cardiovascular research.

## Figures and Tables

**Table 1:** Descriptive and reliability statistics for long-axis right ventricular thickness.

Right Ventricular Thickness (Long Axis)	Mean (SD)	Median (IQR)	Range (Min–Max)	ICC	Residual SD	CV (%)
Systolic (mm)	0.43 (0.05)	0.43 (0.07)	0.31–0.56	0.390	0.04	9.1
Diastolic (mm)	0.43 (0.04)	0.43 (0.04)	0.33–0.55	0.137	0.04	8.2

**Table 2:** Descriptive and reliability statistics for short-axis right ventricular thickness.

Right Ventricular Thickness (Short Axis)	Mean (SD)	Median (IQR)	Range (Min–Max)	ICC	Residual SD	CV (%)
Systolic (mm)	0.46 (0.07)	0.46 (0.11)	0.32–0.69	0.776	0.04	7.9
Diastolic (mm)	0.45 (0.07)	0.45 (0.09)	0.31–0.62	0.746	0.04	8.1

**Table 3:** Descriptive and reliability statistics for long-axis pulmonary artery diameter.

Pulmonary Artery Diameter (Long Axis)	Mean (SD)	Median (IQR)	Range (Min–Max)	ICC	Residual SD	CV (%)
Systolic (mm)	0.32 (0.07)	0.31 (0.08)	0.17–0.47	0.832	0.03	8.9
Diastolic (mm)	0.29 (0.04)	0.29 (0.05)	0.18–0.40	0.750	0.02	7.5

**Table 4:** Descriptive and reliability statistics for short-axis right ventricular outflow velocity and long-axis pulmonary artery outflow velocity.

Outflow Velocities	Mean (SD)	Median (IQR)	Range (Min–Max)	ICC	Residual SD	CV (%)
Right Ventricular Outflow Velocity (mm/s)	177.92 (16.48)	175.43 (23.11)	145.81–218.54	0.805	8.16	4.6
Pulmonary Artery Outflow Velocity (mm/s)	132.14 (20.66)	126.99 (34.24)	95.15–166.89	0.899	6.81	5.2

## Data Availability

Data is included in the manuscript. Raw data can be requested from the corresponding author.
